# Supplementary material for: Polymorphism Detection of GDF9 Gene and Its Association with Litter Size in Luzhong Mutton Sheep (Ovis aries)
Source: Animals (Basel). 2021 Feb 22;11(2):571. doi: 10.3390/ani11020571 (PMC7926531; doi:10.3390/ani11020571)
Supplement: Supplementary file 1 [file animals-11-00571-s001.pdf]

>*GDF9* coding region

ATGGCGCTTCCCAACAAATTCTTCCTTTGGTTTTGCTGCTTTGCCTGGCTCTGTTTT
CCTATTAGCCTTGATTCTCTGCCTTCTAGGGGAGAAGCTCAGATTGTAGCTAGGAC
TGCGTTGGAATCTGAGGCTGAGACTTGGTCCTTGCTGAACCATTTAGGTGGGAGA
CACAGACCTGGTCTCCTTTCCCCTCTCTTAGAGGTTCTGTATGATGGGCACGGGGA
ACCCCCCAGGCTGCAGCCAGATGACAGAGCTTTGCGCTACATGAAGAGGCTCTAT
AAGGCATACGCTACCAAGGAGGGGACCCCTAAATCCAACAGACGCCACCTCTAC
AACACTGTTCGGCTCTTCACCCCCTGTGCTCAGCACAAGCAGGCTCCTGGGGACC
TGGCGGCAGGTGTGTAGGAGCAGATTGGTTAATGGGTGGAGGGAAGAAGAAAGA
CCTTTTTGCATTTCAGTTACATAAAGGAGTTGGCCCTGCTCCTTGACTTGCATTTTA
CTTTGCATGGTACTCAATATCCAAACAAACCTGGTGCTTGATCTTACTGTTTATTCC
TAATGCCCTCATGGGTTGATGTAGGCTAAATCTCTTGCTAGAAGAGATTTAATGGC
CTTAAAACCTGGGAAGAGTGCTGCTGGAGGTTAGCATCTTGTCCAGGGTCACAAA
ATCAAGACTGTAAAAAAAGGTATTTTGACTTCGTTGCATATTCTTTCTAGTCTACCA
ATGTTGGCCTGGACATGAACCTACAGTACCTCTTTCAGCTGTTGGTGTGCTTTAGT
AAACAGGATGTTTTCCCCACGTTTGTTGCTTTCTGTGAGAGGAAAACTGAAGTCC
TTATGTGGAACAGTAGATCACCCTACAAGGACAATGTGTTAATCAACAATCTTGTG
TGTGTGAGAGAGATGGGAGCAGTGCTATTTAAAAAAGAAAAAAAAAATTATATTC
ATTAGCTCGTGACTTACAGAAAACCCTTCTAACCCAGCAGGTCATTTGTGGCTATC
TTAAACCCTTTCTCCTTCCTTGACGTCCTGTCTTGTTACAGTGCGTTTAGAGCTTCC
ATTTGTTGTCAGTCTCAAGTGTTCCAAATTAACTTAGAGAAGTACTTTTTCTTTATT
GATTCTGCCGGTAAGAACTTAGAATGTTTTCTTAACAAGCCTGGCAAGTGTCTTTT
GATAGTTTTCCTCTTAAAGACTTTCCAAGAGAAAAATACTGCTTTCCTTTTTAGCTT
TCTCTTTGTTTGCAGCAACTTAATAAGATTCTAAAACTTCAAAATATAATGATTCCC
TCTTTTAATTCCCCACCAAAGCTATTCTGAATTTCTGGTGATCCAGCAGTAAGTGCT
GGGACTTAGATATGGTACAGATAACTTGCTTTTTCTTTATAACCCCTATCCCACCCT
GACGTTTAAGGCTTGAGAATGTGGGGAGAAAAGGGACAGAAGCACATTCTGAGG
TACTGATTCCTTGATTTGACTTCCTGTTACATATGGCATTACTGTTGGGATTGTTTTT
CTTCTCAGGAACCTTTCCATCAGTGGATCTGCTGTTTAACCTGGATCGTGTTACTGT
TGTGGAACATTTATTCAAGTCAGTTTTGCTATATACTTTCAACAACTCCATTTCTTTT
CCCTTTCCTGTTAAATGTATATGCAACCTGGTGATAAAAGAGCCAGAGTTTTCTAG
CAAGACTCTCCCTAGAGCTCCATACTCATTTACCTATAACTCACAGTTTGAATTTAG
AAAGAAATACAAATGGATGGAGATTGATGTGACGGCTCCTCTTGAGCCTCTGGTG
GCCTCCCACAAGAGGAATATTCACATGTCTGTAAATTTTACATGTGCGAAAGACC
AGCTGCAGCATCCTTCAGCGCGGGACAGCCTGTTTAACATGACTCTTCTCGTAGC
GCCCTCACTGCTTTTGTATCTGAACGACACAAGTGCTCAGGCTTTTCACAGGTGGC
ATTCCCTCCACCCTAAAAGGAAGCCTTCACAGGGTCCTGACCAGAAGAGAGGGC
TATCTGCCTACCCCGTGGGAGAAGAAGCTGCTGAGGGTGTAAGATCGTCCCGTCA
CCGCAGAGACCAGGAGAGTGCCAGCTCTGAATTGAAGAAGCCTCTGGTTCCAGC
TTCAGTCAATCTGAGTGAATACTTCAAACAGTTTCTTTTTCCCCAGAATGAATGTG
AGCTCCATGACTTTAGACTTAGCTTTAGTCAGCTGAAGTGGGACAACTGGATTGTG
GCCCCACACAAATACAACCCTCGATACTGTAAAGGGGACTGTCCCAGGGCGGTC
GGACATCGGTATGGCTCTCCGGTTCACACCATGGTGCAGAACATCATCCATGAGA
AACTTGACTCCTCAGTGCCAAGACCATCCTGTGTACCTGCCAAGTATAGCCCTTTG

AGTGTTTTGGCCATCGAGCCTGATGGCTCAATCGCTTATAAAGAATATGAAGATAT
GATAGCCACTAAGTGTACCTGTCGTTAA
